# Performance of the ForenSeq^TM^ Imagen Kit for Forensic DNA Phenotyping Under Partial Genotyping Conditions

**DOI:** 10.3390/genes17030354

**Published:** 2026-03-23

**Authors:** Nayeli González-Ortiz, Mariano Guardado-Estrada, Nahum Zepeta-Flores, José Miguel Moreno-Ortiz, Adrián Ramírez-de-Arellano, Héctor Rangel-Villalobos, José Francisco Muñoz-Valle, José Alonso Aguilar-Velázquez

**Affiliations:** 1 Laboratorio de Ciencias Morfológico Forenses y Medicina Molecular, Departamento de Morfología, Centro Universitario de Ciencias de la Salud, Universidad de Guadalajara, Guadalajara 44340, Jalisco, Mexico; 2 Doctorado en Genética Humana, Departamento de Biología Molecular y Genómica, Centro Universitario de Ciencias de la Salud, Guadalajara 44340, Jalisco, Mexico; 3 Laboratorio de Genética, Escuela Nacional de Ciencias Forenses, Universidad Nacional Autónoma de México, Ciudad de México 04510, Mexico; 4 Instituto de Genética Humana “Dr. Enrique Corona Rivera”, Departamento de Biología Molecular, Centro Universitario de Ciencias de la Salud, Universidad de Guadalajara, Guadalajara 44340, Jalisco, Mexico; 5 Instituto de Investigación en Cáncer e Infecciones, Departamento de Microbiología y Patología, Centro Universitario de Ciencias de la Salud, Universidad de Guadalajara, Guadalajara 44340, Jalisco, Mexico; 6 Instituto de Investigación en Genética Molecular, Departamento de Ciencias Médicas y de la Vida, Centro Universitario de la Ciénega, Universidad de Guadalajara, Ocotlán 47810, Jalisco, Mexico; 7 Instituto de Investigación en Ciencias Biomédicas, Centro Universitario de Ciencias de la Salud, Universidad de Guadalajara, Guadalajara 44340, Jalisco, Mexico

**Keywords:** forensic DNA phenotyping, ForenSeq imagen kit, performance, prediction

## Abstract

Background: Forensic DNA phenotyping (FDP) enables the inference of externally visible characteristics (EVCs) and biogeographic ancestry when conventional STR profiling is inconclusive. The ForenSeq™ Imagen kit (107 SNPs) integrates phenotype-, ancestry-, and Y-SNPs markers; however, its performance under partial genotyping conditions has not been systematically evaluated. Methods: Ninety-four samples from a Mexican mestizo population were analyzed using the ForenSeq™ Imagen kit on the MiSeq FGx™ platform. Due to incomplete genotype recovery, 41 samples with >60% locus detection were selected for downstream analyses. Phenotype prediction was performed using the HIrisPlex-S model, and ancestry inference was assessed through principal component analysis. In silico simulations were conducted to evaluate locus-specific dropout effects. Results: Eye color prediction showed both reduced feasibility (68.3%) and lower overall accuracy (56.1%), primarily driven by systematic prediction failure when rs12913832 (*HERC2*) was absent, although accuracy among successfully predicted samples remained high (82.1%). In contrast, hair and skin color inference remained feasible in >97% and 100% of evaluable samples, respectively; however, classification accuracy was moderate (70% for hair and 61% for skin), improving substantially when allowing adjacent-category concordance (90.2% for skin). Ancestry inference was robust when at least 27 aiSNPs were detected, and Y-SNPs reliably distinguished male and female samples. In silico analyses confirmed the critical contribution of rs12913832 to eye color model operability. Conclusions: FDP performance under partial genotyping reflects a trade-off between prediction feasibility and accuracy and depends on locus-specific integrity rather than overall genotype completeness. The ForenSeq™ Imagen kit shows robustness for ancestry, sex, hair, and skin prediction, although with variable accuracy, whereas eye color inference remains structurally vulnerable to drop out of high-impact variants. Evaluating FDP systems under realistic non-ideal conditions is essential to define their true operational limits and ensure scientifically robust and responsible implementation.

## 1. Introduction

Since their introduction into forensic genetics, short tandem repeats (STRs) have become the markers of choice for human identification. Their high polymorphism and strong discriminatory power have enabled reliable individualization across a broad range of forensic contexts. Nevertheless, despite their extensive utility, STR-based approaches present important limitations. These include reduced performance in cases involving degraded or low-quality template DNA, susceptibility to stochastic effects such as allele drop-out, and limited applicability in scenarios where no reference samples are available for comparison, as is frequently the case in unidentified human remains or investigative casework [1].

In such contexts, single nucleotide polymorphisms (SNPs) have emerged as valuable complementary markers. Due to their biallelic nature and short amplicon size, SNPs are particularly suitable for degraded DNA samples. Moreover, depending on their genomic location and functional relevance, certain SNPs enable the inference of biogeographic ancestry (ancestry-informative SNPs, aiSNPs) as well as externally visible characteristics (phenotype-informative SNPs, piSNPs), including eye, hair, and skin color, among other externally visible characteristics (EVCs) [2].

The inference of EVCs from DNA, commonly referred to as Forensic DNA Phenotyping (FDP), has gained increasing relevance in forensic investigations where conventional identification methods are insufficient. FDP is particularly useful when physical descriptors of the DNA donor are unavailable, such as in cases involving highly decomposed remains or when STR profiles obtained from crime scene samples fail to generate database matches. In these situations, FDP can provide investigative leads by narrowing the pool of potential contributors and guiding casework prioritization [1].

Several SNP-based systems have been developed specifically for FDP. Early examples include IrisPlex for eye color prediction [3], followed by HIrisPlex, which expanded phenotype inference to include hair color [4], and HIrisPlex-S, which further incorporates skin color prediction [5]. As the number of markers required for comprehensive phenotype inference increased, massively parallel sequencing (MPS) technologies were introduced to forensic genetics, enabling the simultaneous analysis of large SNP panels across multiple samples within a single assay [6]. Currently, the most widely used MPS platforms in forensic research are Ion Torrent (Thermo Fisher Scientific, Waltham, MA, USA) and the MiSeq (Illumina, San Diego, CA, USA) platforms [2].

Several commercial MPS-based kits for FDP have been validated and implemented in forensic research and practice. Among these, the ForenSeq™ DNA Signature Prep Kit with DNA Primer Set B (DPSB) enables the inference of eye and hair color phenotypes and has been extensively evaluated across diverse populations, with studies consistently reporting high prediction accuracy for blue and brown eye color, as well as blond and red hair color [7,8,9,10].

More recently, the ForenSeq™ Imagen kit (Illumina, San Diego, CA, USA) was developed as an integrated genomic system comprising 107 SNPs distributed across three marker panels: (i) 41 piSNPs for eye, hair, and skin color inference; (ii) 56 aiSNPs for biogeographic ancestry estimation; and (iii) 14 Y-SNPs for sex determination. Despite its potential forensic utility, there are currently no comprehensive validation studies assessing the kit’s performance for its intended forensic applications. The only published study involving this system explored its use for predicting EVCs beyond the scope for which the kit was designed [11] and has been subject to substantial methodological criticism regarding study design and result interpretation [12,13].

The evaluation and implementation of FDP technologies such as the ForenSeq™ Imagen kit are particularly relevant in countries like Mexico, where forensic identification faces significant challenges. According to official records, as of September 2023, a total of 294,297 cases have been documented, including 111,516 (37.9%) missing persons and 182,781 (62.1%) located individuals since 1962 [14]. A considerable proportion of unidentified individuals remain unresolved due to degraded biological material or the absence of first-degree relatives for comparative genetic analysis, thereby limiting the effectiveness of conventional STR-based identification approaches [15].

In this context, the ForenSeq™ Imagen kit represents a promising MPS-based tool that integrates multiple informative SNP panels within a single assay. The present study aims to evaluate the applicability and operational performance of this system in a Mexican population, with particular emphasis on its accuracy for forensic DNA phenotyping under conditions of partial genotype recovery. By assessing its performance in realistic forensic laboratory conditions, this work seeks to contribute to the implementation of FDP technologies as complementary tools for human identification and to help reduce the current backlog of unidentified human remains.

## 2. Materials and Methods

### 2.1. Population Sample

A total of 94 genomic DNA samples were obtained from unrelated adult volunteers residing in Jalisco, Mexico. Participants ranged in age from 18 to 38 years and included 72 males and 22 females. Written informed consent was obtained from all individuals prior the enrollment. The study was conducted in accordance with established ethical standards for research involving human subjects and was approved by the Research Ethics Committee (Comité de Ética en Investigación (CEI) from the Centro Universitario de Ciencias de la Salud of the Universidad de Guadalajara; approved code CI-06824).

### 2.2. Reference Phenotype Assignment

High-resolution digital photographs were obtained using a Canon EOS Rebel T6 camera (Canon Inc., Tokyo, Japan). Images included close-up photographs of the iris, the posterior scalp for hair color assessment, and the forearm for skin pigmentation evaluation. All images were compiled into a standardized Microsoft Excel for Microsoft 365 (Microsoft Corporation, Redmond, WA, USA) database.

Three independent analysts evaluated each volunteer’s phenotype based on the photographic documentation. Phenotypic categories were assigned according to classification schemes compatible with the HIrisPlex-S prediction system. Phenotypes were categorized as follows: (A) Eye color: blue, intermediate (green, hazel, and yellowish tones), and brown. (B) Hair color: blond, red, brown, and black, with light or dark shades. (C) Skin color: Fitzpatrick phototypes I (very pale), II (pale), III (intermediate), IV (dark), and V (very dark/black). Consensus was achieved in all cases across the three evaluators. These assigned phenotypes were used as reference data for subsequent assessments of prediction accuracy.

### 2.3. DNA Extraction and Quantification

Genomic DNA was extracted from peripheral blood using a CTAB/DTAB method based on the protocol described by Miller [16]. This protocol employs the cationic detergents cetyltrimethylammonium bromide (CTAB) and dodecyltrimethylammonium bromide (DTAB) to lyse cells, remove proteins and other contaminants, and selectively precipitate high-molecular-weight DNA. The resulting DNA was stored at −20 °C until further analysis. Extracted DNA was quantified by fluorometric analysis using the Qubit™ 4 Fluorometer (Thermo Fisher Scientific, Waltham, MA, USA), following the manufacturer’s instructions.

### 2.4. Library Preparation and Massively Parallel Sequencing

Genomic libraries were prepared using the ForenSeq™ Imagen kit (Verogen Inc., San Diego, CA, USA) according to the manufacturer’s protocol. Library preparation began with an input of 125 pg of genomic DNA. Target regions were amplified and tagged during an initial PCR step, followed by a second enrichment PCR incorporating Unique Dual Index (UDI) sequences and sequencing adapters.

Following amplification, libraries were purified enzymatically using magnetic bead-based cleanup procedures to remove residual primers and reaction components. Libraries were subsequently normalized to achieve equimolar concentrations before pooling. Equal volumes of each normalized library were combined to generate a pooled library mixture.

The initial 94 libraries were pooled, diluted in hybridization buffer, and heat-denatured before being loaded onto the MiSeq FGx micro flow cell for a single run (Verogen Inc., San Diego, CA, USA). Sequencing was performed according to the MiSeq FGx™ System Reference Guide (Verogen Inc., San Diego, CA, USA). The sequencing run included both positive and negative controls to monitor amplification performance and potential contamination.

### 2.5. Evaluation of Sequencing Run Quality

Sequencing run quality was assessed using post-sequencing performance and quality metrics generated by the MiSeq FGx™ platform. The following parameters were evaluated to determine overall sequencing efficiency and data reliability. First, cluster density was examined and expressed as the number of clusters per square millimeter (K/mm^2^). For the Forensic Imagen kit, the recommended optimal range is between 400 and 1650 K/mm^2^, which reflects appropriate library loading and cluster generation efficiency. Second, clusters passing filters (PFs) were evaluated as the percentage of total clusters that met Illumina’s quality filtering criteria. This metric reflects the proportion of clusters with sufficient signal intensity and base-calling reliability. A PF value of ≥80% was considered indicative of acceptable sequencing performance.

Third, phasing and prephasing rates were assessed as indicators of synchronization efficiency during the sequencing-by-synthesis process. Phasing represents the percentage of DNA molecules within a cluster that lags the current sequencing cycle due to incomplete nucleotide incorporation, whereas prephasing corresponds to the proportion of strands that advance of the cycle because of premature incorporation events. In accordance with the manufacturer’s recommendations, acceptable thresholds were defined as ≤0.25% for phasing and ≤0.15% for prephasing.

In addition, overall read quality was evaluated using Illumina’s per-cycle quality score visualization, which summarizes average base-call quality across sequencing cycles using a color-coded system. In this system, green indicates that the mean quality scores fall within the expected performance range, whereas orange denotes deviations from optimal sequencing quality. These metrics were used to evaluate the technical quality of each sequencing run to ensure data reliability before genotyping and phenotype inference analyses.

### 2.6. Definition of Partial Genotypes and Profile Inclusion Criteria

A partial genotype was defined as a genetic profile in which one or more marker sets failed to produce complete SNP data but retained sufficient genetic information to support phenotype or ancestry inference. Profiles were considered suitable for downstream analyses when genotype data allowed for successful processing within the HIrisPlex-S prediction framework or ancestry assessment via principal component analysis (PCA). Samples lacking sufficient genotype information to generate any phenotype or ancestry inference were excluded from performance evaluations but retained for overall sequencing outcome reporting.

### 2.7. Genotype Processing and Data Management

Genotype data generated by the MiSeq FGx™ instrument were processed using Verogen’s Universal Analysis Software (UAS) version 1.3 (Verogen Inc., San Diego, CA, USA). Genotyping results, sequencing metrics, and locus-level information were exported in Microsoft Excel format for subsequent analyses. Missing SNP calls were retained as missing data and were not imputed or manually corrected. Downstream predictive analyses were performed using the genotype information available for each sample, without additional filtering or data inference.

### 2.8. Forensic DNA Phenotyping Analysis

Phenotype prediction was performed using the HIrisPlex-S web-based tool developed by Erasmus Medical Center (https://hirisplex.erasmusmc.nl/, accessed on 12 February 2025). This system predicts eye, hair, and skin color using validated SNP panels and a prediction model trained on diverse continental populations [3,4,5]. The R script provided by the EMC platform was used to convert piSNP output files generated by UAS into the required input format for phenotype prediction. Predictions were generated for all samples that met the profile inclusion criteria.

Predicted phenotypes were compared with reference phenotypes assigned from photographic analysis. Prediction performance was assessed by calculating the percentage of correctly classified samples for each phenotypic trait. Additionally, the number of SNPs contributing to successful phenotype prediction and the number of genotypes producing interpretable predictions were recorded to evaluate system performance under conditions of partial genotype recovery and incomplete marker representation.

To evaluate the relative contribution of individual eye color SNPs to prediction feasibility, an in silico dropout analysis was performed using the HIrisPlex-S online platform (Erasmus Medical Center). Complete genotype profiles were systematically modified by artificially removing one SNP at a time from the IrisPlex eye color panel while retaining all remaining loci. Six independent simulations were conducted, each corresponding to the exclusion of a different eye color SNP. The modified datasets were reanalyzed to determine whether phenotype prediction remained viable in the absence of each specific marker. This approach allowed for the assessment of locus-specific sensitivity within the predictive model under controlled virtual dropout conditions.

### 2.9. Biogeographic Ancestry Analysis

Biogeographic ancestry inference was evaluated by visual inspection of sample clustering patterns in the principal component analysis (PCA) generated by the Universal Analysis Software (UAS) v2.x software (Verogen, San Diego, CA, USA). Samples were assessed based on their relative positions within PCA space and their proximity to reference population clusters included in the analysis. No formal ancestry assignment thresholds were applied.

### 2.10. Statistical Approach

All analyses were descriptive and were designed to evaluate operational system performance, profile recovery, and interpretability of phenotype and ancestry predictions under partial genotyping conditions. No inferential statistical testing was performed, as the primary objective was to assess methodological performance in realistic laboratory forensic scenarios.

## 3. Results

### 3.1. Sequencing Run Quality

Bioinformatic analysis was performed using UAS v2 with the ForenSeq™ Imagen Geo assay. The sequencing run comprised four reads (Read 1, Index 1, Index 2, and Read 2), totaling 318 cycles. All evaluated run metrics were within the manufacturer’s recommended thresholds, indicating appropriate sequence performance and data reliability.

The cluster density was 478 K/mm^2^, falling within the optimal recommended range (400–1650 K/mm^2^). A total of 98.09% of clusters passed Illumina’s quality filter (PF), exceeding the minimum acceptable threshold of 80% and demonstrating high signal quality and base-calling reliability.

Phasing and prephasing values were 0.205% and 0%, respectively, both within the acceptable limits (≤0.25% for phasing and ≤0.15% for prephasing), indicating adequate synchronization during the sequencing-by-synthesis process without compromising read accuracy. Per-cycle quality score plots showed mean base-call quality values within the expected (green) performance range across all sequencing cycles. No discordant genotypes were observed across loci, indicating stable assay performance under the evaluated sequencing conditions.

According to the reference manual, the assay can generate up to 50,000 reads per sample. In this run, the number of reads varied substantially across samples, ranging from 148 to 90,000. Given this variability, the performance of the HSC control was considered critical for determining the inclusion of samples with low read counts in subsequent analyses under partial genotyping conditions.

### 3.2. Genotype Recovery and Profile Completeness

A total of 94 samples were processed using the ForenSeq™ Imagen kit, targeting 41 phenotype-informative SNPs (piSNPs), 56 ancestry-informative SNPs (aiSNPs), and 14 Y-SNPs, for a total of 107 markers. Of these, 53 samples (56.4%) were genotyped for only 5–57 markers and produced between 148–29,000 total reads; consequently, they were excluded from the analyses presented in this study. In contrast, 41 samples (43.6%) achieved a genotyping rate greater than 60% of the total panel and were considered suitable for downstream phenotype and ancestry inference analyses (Table S1 and Table 1). Although these samples yielded partial genetic profiles, they retained sufficient marker representation to support phenotype and ancestry inference.

Within the piSNP panel (41 markers), locus recovery varied across trait categories. For the eye color subpanel (6 SNPs), between 3 and 6 loci were successfully genotyped per sample. The rs12913832 (*HERC2*) SNP was successfully genotyped in 28 out of 41 samples (68.3%), while dropout was observed in 13 samples (31.7%). In 13 samples, eye color prediction was not possible. In all of these cases, amplification failure of rs12913832 was observed. Samples 124, 153, and 154 amplified only 4, 4, and 3 eye-related SNPs, respectively. In the remaining 10 samples where prediction was not possible (08, 19, 23, 25, 31, 47, 96, 122, 163, and 303), five SNPs were successfully genotyped; however, rs12913832 was consistently absent. Notably, other samples (e.g., 74 and 88) also showed five successfully genotyped SNPs but retained rs12913832, allowing phenotype prediction. This pattern highlights the critical role and predictive dependency of rs12913832 within the HIrisPlex-S eye color prediction model.

For the hair color panel (22 SNPs), between 12 and 22 loci were successfully genotyped across samples. Phenotype inference was achieved in 40 out of 41 samples (97.6%). Only sample 147, which presented 11 genotyped SNPs, did not yield a hair color prediction. Regarding the skin color panel (36 SNPs), a minimum of 25 loci were successfully genotyped in all 41 samples. In all cases, sufficient genetic information was available to generate a phenotype prediction (100% prediction feasibility).

For the 56 aiSNPs, at least 27 markers were successfully genotyped per sample, which was sufficient to generate principal component analysis (PCA) plots for ancestry inference in all evaluable cases. Concerning the Y-SNP panel, Y markers were not detected in 10 samples, corresponding to female individuals. The presence of at least one Y-SNP was sufficient to determine male sex in the remaining samples.

### 3.3. Phenotype Prediction Performance Under Partial Genotyping Conditions

Phenotype predictions obtained using the HIrisPlex-S system were compared against the observed phenotypes derived from standardized photographic assessment in the 41 samples that generated partial but interpretable genotypes (Table 2). Performance was evaluated using prediction rate (the proportion of samples for which a prediction was generated), strict classification accuracy, overall accuracy (including failed predictions), and balanced performance across categories.

Eye color prediction was successfully generated in 28 out of the 41 samples (68.3%). In the remaining 13 samples (31.7%), no prediction could be generated due to insufficient marker recovery. Notably, all failed predictions shared the absence of rs12913832, confirming its pivotal role within the HIrisPlex-S model. Among the 28 samples with available predictions, 23 were correctly classified and 5 were misclassified, yielding a strict classification accuracy of 82.1% among the predicted samples. When failed predictions were included, the overall accuracy decreased to 56.1%. Misclassifications primarily involved confusion between blue and brown eye color categories, as well as the assignment of intermediate eye color to the brown category. No extreme or biologically implausible classifications were observed. The relatively high accuracy between predicted samples suggests that once the core SNPs—particularly rs12913832—are recovered, the system retains strong discriminatory capacity even under partial genotyping conditions. However, the reduced prediction rate highlights the sensitivity of eye color inference to locus dropout.

Hair color prediction was achieved in 40 out of 41 samples (97.6%), demonstrating high robustness to partial genotype recovery. Only one sample failed to generate a prediction due to insufficient SNP recovery (11 of 22 loci). Among the 40 predicted samples, 28 were correctly classified, and 12 were misclassified, corresponding to a strict classification accuracy of 70.0% and an overall accuracy of 68.3%. Most discrepancies occurred between adjacent pigmentation categories, particularly between black and dark brown, and between brown and blond. Importantly, the single red-haired individual was correctly predicted, consistent with the high discriminatory performance of the red hair-associated markers. The lower accuracy relative to eye color likely reflects the polygenic complexity of hair pigmentation and the continuous variation observed in admixed populations, where categorical classification may oversimplify biological reality. Balanced performance across hair color categories revealed reduced sensitivity in distinguishing dark shades, suggesting that partial marker recovery may disproportionately affect the resolution of closely related pigmentation phenotypes.

Skin color prediction was successfully generated in all 41 samples (100% prediction rate), indicating strong resilience of the 36-marker panel to partial genotype conditions. Strict classification accuracy was 61.0% (25/41). However, when allowing a one-category deviation (e.g., phototype II predicted as III), concordance increased substantially to 90.2% (37/41). No extreme misclassifications were observed. Most discrepancies occurred between adjacent phototypes (II–III and III–IV), consistent with the continuous distribution of pigmentation in admixed populations. These findings suggest that although categorical precision may decrease under partial genotyping, the system maintains high biological plausibility and practical utility for investigative purposes.

### 3.4. In Silico Sensitivity Assessment of the IrisPlex Eye Color Panel

Considering the eye color prediction failures observed under partial genotyping conditions, an additional in silico sensitivity analysis was conducted to evaluate the relative contribution of each SNP within the IrisPlex eye color panel to model operability.

Six independent simulation assays were performed using the HIrisPlex-S online platform. For each assay, complete genotype datasets were artificially modified to introduce systematic locus-specific dropout across all individuals. In the first simulation, genotypes at rs12913832 were removed for the entire sample set. In subsequent simulations, dropout was sequentially introduced at rs1800407 and each of the remaining IrisPlex eye color SNPs, one at a time, while keeping all other loci intact. This approach allowed the isolated evaluation of each marker’s contribution to prediction feasibility.

The simulations demonstrated that eye color prediction remained possible for all individuals as long as rs12913832 was retained in the genotype profile. In contrast, artificial removal of rs12913832 resulted in complete prediction failure across the dataset, regardless of the presence of the remaining SNPs. Dropout of any other individual marker did not prevent the generation of eye color predictions.

These findings confirm that rs12913832 functions as the primary driver of model operability within the IrisPlex system. Its absence compromises the algorithm’s capacity to compute posterior probabilities, explaining the systematic prediction failures observed in the empirical partial genotypes described above. The in silico results, therefore, provide mechanistic support for the experimental data and highlight the disproportionate weight of rs12913832 in eye color inference.

### 3.5. Comparative Robustness Across Traits

When comparing performance metrics across traits, clear differences in robustness under partial genotyping conditions were observed. Skin color inference demonstrated the highest resilience, with a 100% prediction rate and high adjacent-category concordance. Hair color prediction also showed high feasibility (97.6%), although with moderate classification accuracy. In contrast, eye color prediction was the most sensitive to marker dropout, primarily driven by the absence of rs12913832, resulting in the lowest prediction rate (68.3%) despite high accuracy among successfully predicted samples. These results indicate that the predictive stability of the ForenSeq™ Imagen kit under partial genotyping conditions is trait-dependent and strongly influenced by the relative weight of individual SNPs within each predictive model.

### 3.6. Biogeographic Ancestry Inference

**Figure 1:**
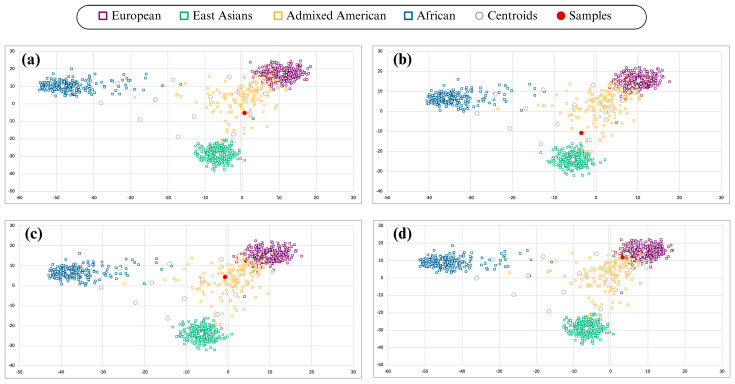
Distribution of ancestry patterns in 41 samples analyzed using the ForenSeq™ IMAGEN kit. The following groups were observed: (**a**) centroid pattern of American Mestizos; (**b**) pattern slightly shifted toward East Asian ancestry; (**c**) pattern with a slight shift toward European ancestry; and (**d**) pattern strongly shifted toward European ancestry.

All 41 samples yielded sufficient aiSNP recovery (≥27 of 56 loci) to generate interpretable principal component analysis (PCA) clustering patterns. The observed distributions revealed four predominant ancestry patterns (Figure 1): (a) centroid with predominantly American ancestry (admixed); (b) slightly inclined towards the East Asian population, probably showing higher Native American ancestry; (c) slightly inclined towards the European sample; and (d) highly inclined towards the European sample, showing individuals with high European ancestry. Interestingly, samples with the European pattern were the most frequent (46.3%), while the East Asian/Native American pattern was the least frequent (7.3%). Partial genotype recovery did not preclude ancestry inference in any sample, indicating that the aiSNP panel retains analytical stability even when full marker recovery is not achieved.

## 4. Discussion

### 4.1. Technical Performance Under Partial Genotyping Conditions

This study, to our knowledge, provides the first independent evaluation of the ForenSeq™ Imagen kit under partial genotyping conditions, offering insight into system behavior beyond ideal validation settings. Although complete genotype recovery was not achieved across all samples, the partial profiles generated allowed for the assessment of the operational tolerance of phenotype and ancestry inference models when confronted with incomplete genetic information—an increasingly realistic scenario in forensic casework involving degraded or limited DNA [1].

A considerable proportion of samples exhibited locus-specific dropout across different marker panels. Importantly, the results demonstrate that prediction feasibility is not directly proportional to the overall percentage of recovered loci. Instead, model operability depends disproportionately on retaining specific high-impact variants. This distinction is critical: global genotyping success rates may appear acceptable while phenotype prediction fails due to the absence of a single pivotal marker.

The spatial clustering of dropout events across the sequencing plate suggested a technical origin associated with multichannel pipetting variability during library preparation. Recurrent amplification failure in samples occupying the same channel position supports the hypothesis of uneven reagent dispensing. Such pre-analytical variability is consistent with high-throughput MPS workflows and has been recognized as a source of locus imbalance in forensic MPS applications [7]. These findings suggest that the incomplete genotype recovery observed in this study likely reflects workflow-related variability rather than intrinsic limitations of the ForenSeq™ Imagen kit chemistry. Studies evaluating related MPS-based forensic workflows have also reported locus imbalance and variable marker recovery when analyzing degraded or otherwise challenging DNA samples, particularly under degraded conditions [17]. Nonetheless, the downstream interpretive consequences remain operationally relevant.

An additional factor that may have contributed to the observed variability in genotyping performance is the absence of a human-specific DNA quantification method. In forensic NGS workflows, qPCR-based quantification assays targeting human DNA are critical to accurately estimate the amount of amplifiable template. Non-specific quantification methods may overestimate DNA concentration in the presence of degradation, non-human DNA, or inhibitors, potentially leading to suboptimal library input and increased allele dropout. Therefore, the incomplete genotype recovery observed in this study may be partially explained by limitations in DNA quantification, in addition to stochastic and technical factors during library preparation.

Importantly, the overall sequencing run quality metrics were well within the manufacturer’s recommended thresholds, including optimal cluster density, high percentages of clusters passing filters, and acceptable phasing and prephasing rates. Per-cycle quality plots confirmed stable base-calling performance throughout the run. These findings indicate that the locus-specific dropout observed in this study cannot be attributed to suboptimal sequencing chemistry or instrument performance, reinforcing the interpretation that pre-analytical workflow variability is the primary contributing factor.

### 4.2. Phenotype Prediction Robustness and Marker-Specific Sensitivity

Eye color prediction was critically dependent on the presence of rs12913832. Both empirical observations and in silico dropout simulations consistently showed that its absence led to systematic prediction failure, even when the remaining IrisPlex markers were successfully genotyped. This behavior is consistent with the design of the HIrisPlex-S model, which does not generate eye color predictions when rs12913832 is missing [5]. In contrast, dropout affecting other individual SNPs did not prevent phenotype inference.

The relatively high frequency of rs12913832 dropout observed in this study further explains the reduced feasibility of eye color prediction under partial genotyping conditions. This dependency reflects the biological and statistical architecture of pigmentation models. The rs12913832 variant, located within an intronic enhancer region of HERC2, regulates OCA2 expression and exerts a major effect on blue versus brown eye pigmentation [3,18,19]. Its strong predictive weight explains why HIrisPlex-S operability collapses when this marker is absent. Thus, the observed sensitivity does not represent dataset-specific instability but rather an inherent structural characteristic of the model.

Intermediate eye colors showed greater variability, consistent with previous IrisPlex validation studies reporting reduced accuracy for non-binary pigmentation categories [3,5]. This limitation is particularly relevant in admixed populations such as Mexican mestizos, whose tri-hybrid ancestry components (European, Native American, and African) generate continuous ancestry and pigmentation gradients that challenge models trained predominantly on European reference datasets [3,5,20,21].

In contrast, hair and skin color prediction exhibited greater resilience under partial genotype recovery. Hair prediction remained feasible in over 97% of samples despite recovery ranging from 12 to 22 SNPs, while skin color inference was successful with as few as 25 of 36 loci. The polygenic architecture of these traits, involving distributed effect sizes across multiple loci [5], likely confers greater tolerance to partial genotype loss compared with the near single-locus dependency observed for eye color.

### 4.3. Ancestry and Y-SNP Inference Stability

Biogeographic ancestry inference demonstrated notable robustness. All samples with at least 27 aiSNPs produced interpretable PCA clustering patterns. This resilience likely reflects the multivariate structure of ancestry inference, where population differentiation signals are distributed across numerous loci rather than concentrated in a single variant [1]. Unlike eye color prediction, which can collapse with the loss of a critical SNP, ancestry inference relies on cumulative allele frequency patterns across populations.

Similarly, Y-SNP detection reliably distinguished male and female samples, as no Y-SNPs were detected in female individuals. The presence of even a single Y-SNP was sufficient for sex determination, confirming analytical specificity despite incomplete recovery. The utility of Y-linked markers for sex determination in forensic genetics has been widely established [22].

### 4.4. Comparison with Previous Mexican MPS-Based FDP Studies

Previous studies conducted in Mexican populations using the ForenSeq™ DNA Signature Prep kit (DPSB configuration) reported high accuracy in eye color prediction, particularly for brown phenotypes (>96%), and variable performance for hair color depending on the analytical tool used. Those studies, however, were performed under conditions of near-complete genotype recovery [10,23].

The present work extends these findings by examining system behavior under incomplete marker recovery. While the previous works demonstrated the feasibility of FDP in Mexican mestizo cohorts, they did not explicitly evaluate the impact of locus-specific dropout on prediction operability. Our results indicate that even modest marker loss may critically impair prediction if high-impact loci such as rs12913832 are absent.

Moreover, the predominance of brown eyes and dark hair in Mexican cohorts [10,23] may inflate classification accuracy for majority phenotypes while limiting the evaluation of rarer categories. Under partial genotyping conditions, these structural dependencies become more evident.

Compared to exploratory analyses of the ForenSeq™ Imagen kit for additional phenotypes [11], which focused on trait expansion rather than operational robustness, the present study provides performance-based evidence regarding genotype completeness thresholds and marker dependency, an aspect essential for real forensic implementation.

### 4.5. Practical Implications for Forensic Casework

An important operational insight emerging from this study is that the reliability of phenotype prediction cannot be inferred solely from overall genotyping percentages. Instead, locus-specific integrity must be evaluated. Laboratories implementing MPS-based forensic DNA phenotyping systems should monitor marker-level recovery metrics and transparently report missing high-impact loci.

Although the strong association between rs12913832 and eye color is well-established [3,18,19], the present results highlight an important operational implication for forensic DNA phenotyping workflows. When genotype profiles are incomplete, the absence of this single high-impact SNP can prevent reliable eye color prediction, even if most other loci are successfully recovered. Therefore, locus-level quality assessment becomes critical during data interpretation. Laboratories implementing MPS-based FDP systems should explicitly verify and report the recovery of key predictive markers such as rs12913832 when presenting phenotype inference results derived from partial genotype profiles.

In countries such as Mexico, where large numbers of unidentified remains persist, and reference samples are often unavailable [24], FDP may provide valuable investigative leads. However, predictions derived from incomplete profiles must be interpreted with caution. Overinterpretation of predictions in the absence of critical markers should be avoided to ensure the responsible application of forensic analysis.

### 4.6. Study Limitations and Future Directions

This study has some limitations that should be considered when interpreting the results. First, the analyses were performed using reference samples under controlled laboratory conditions rather than typical forensic casework material. Consequently, the DNA analyzed in this study does not fully replicate the range of degradation patterns commonly encountered in forensic investigations. In this context, the genotype loss observed across some samples likely reflects technical variability during library preparation rather than environmental DNA damage or other common forensic challenges. Additionally, the number of evaluable partial genotypes was moderate, and genotype loss was not systematically evaluated using controlled degradation or low-template DNA experiments. Therefore, quantitative thresholds for minimum DNA input or degradation tolerance could not be defined.

Another limitation concerns the ancestry inference analysis. In this study, population structure was explored using principal component analysis (PCA), and ancestry patterns were interpreted based on visual clustering of the samples relative to reference populations. While PCA is widely used as an exploratory tool in population genetics, this approach does not provide probabilistic ancestry assignment, admixture proportions, or formal classification accuracy estimates. Consequently, the ancestry interpretations presented here should be considered descriptive rather than definitive.

Future research should incorporate probabilistic ancestry inference approaches, such as admixture-based models or assignment tests, to provide quantitative estimates of ancestry proportions and classification probabilities. However, this task appears to be difficult in the short-term, as no databases of Mexican Native American groups have been reported that are compatible with the aiSNPs included in this kit, a prerequisite for determining percentages of ancestry and genetic admixture [25].

Additionally, future studies should incorporate controlled degradation models, low-template DNA experiments, and samples containing potential inhibitors to better simulate realistic forensic conditions. Replicate library preparations could also be performed to assess technical reproducibility, and quantitative analyses should be implemented to determine the minimum number of loci required for stable phenotype and ancestry prediction across traits.

## 5. Conclusions

Collectively, these findings demonstrate that the ForenSeq™ Imagen kit retains practical utility under partial genotyping conditions, particularly for hair color, skin color, ancestry, and sex inference. However, eye color prediction exhibits structural vulnerability due to its dependence on rs12913832, a locus consistently identified as the principal determinant in IrisPlex-based systems. Evaluating forensic DNA phenotyping systems under realistic non-ideal conditions is essential to define their true operational limits and ensure scientifically robust and responsible implementation.

## Figures and Tables

**Table 1:** Number of markers successfully amplified in each panel for every individual in the study population. The numbers of loci included in each panel are indicated in parentheses.

Sample	piSNP				aiSNP (56)	Y-SNP (14)	Total (107)
	Eye (6)	Hair (22)	Skin (36)	Subtotal (41)			
08	5 *	19	31	35	40	F	75
19	5 *	20	31	36	42	11	89
23	5 *	20	31	36	39	F	75
24	6	22	34	39	47	12	98
25	5 *	16	29	32	38	F	70
31	5 *	21	32	37	41	F	78
34	6	20	32	36	42	12	90
47	5 *	19	30	33	40	10	83
66	6	22	35	40	52	14	106
74	5	16	29	32	50	10	92
81	6	22	35	40	54	12	106
82	6	22	35	40	55	14	107
83	6	22	35	40	52	12	104
88	5	17	30	33	47	8	88
89	6	21	32	37	44	F	81
91	6	22	34	39	51	12	102
96	5 *	20	31	36	35	10	81
98	6	22	35	40	51	12	103
103	6	22	35	40	54	14	107
104	6	22	36	41	53	12	106
106	6	21	34	39	48	12	99
107	6	22	35	40	52	12	104
109	6	21	31	37	44	11	92
122	5 *	20	32	37	40	11	88
123	6	21	32	37	44	F	81
124	4 *	14	27	30	36	8	74
134	6	21	33	38	48	11	97
144	6	22	34	39	50	12	101
145	6	22	33	38	42	F	80
147	6	11*	23	24	47	6	77
151	6	22	35	40	52	F	92
152	6	22	35	40	51	F	91
153	4 *	13	25	27	29	7	63
154	3 *	12	25	27	27	6	60
155	6	22	35	40	51	12	103
156	5 *	20	31	36	44	F	80
159	6	22	35	40	53	13	106
160	6	22	35	40	53	12	105
158	6	22	35	40	48	F	88
254	6	22	34	39	49	12	100
303	5 *	15	29	31	36	8	75

F: female sample, no Y-SNPs detected; * samples where no prediction was possible.

**Table 2:** Observed and predicted phenotypes, including their corresponding area under the curve (AUC) values. The following categories were considered: eye color (brown, blue, or intermediate); hair color (brown, black, blond, or red); and skin color classified as very pale or phototype I (FI), pale or phototype II (FII), intermediate or phototype III (FIII), dark or phototype IV (FIV), and black or phototype V (FV). NA was used for non-generated data.

	Eye Color			Hair Color			Skin Color		
Sample	Observed	Predicted (p)	AUC	Observed	Predicted (p)	AUC	Observed	Predicted (p)	AUC
8	Brown	NA	0.9460	Brown	Black (0.5194)	0.8592	F-III	F-III (0.9432)	0.7833
19	Brown	NA	0.9460	Brown	Black (0.5886)	0.8592	F-III	F-III (0.9998)	0.7833
23	Intermediate	NA	0.9460	Brown	Black (0.7216)	0.8592	F-II	F-III (0.4844)	0.7833
24	Brown	Brown (0.9909)	0.9460	Brown	Brown (0.8201)	0.7410	F-III	F-III (0.9999)	0.7833
25	Brown	NA	0.9460	Brown	Blond (0.5128)	0.8132	F-II	F-III (0.7337)	0.7833
31	Brown	NA	0.9460	Brown	Blond (0.5020)	0.8132	F-II	F-III (0.9995)	0.7833
34	Brown	Brown (0.9946)	0.9460	Black	Black (0.6699)	0.8592	F-IV	F-III (0.9952)	0.7833
47	Brown	NA	0.9460	Black	Brown (0.4771)	0.7410	F-III	F-III (0.9543)	0.7833
66	Brown	Brown (0.9930)	0.9460	Black	Brown (0.6974)	0.7410	F-III	F-III (0.9992)	0.7833
74	Brown	Brown (0.9784)	0.9460	Black	Brown (0.5391)	0.7410	F-III	F-V (0.9540)	0.9934
81	Brown	Brown (0.9117)	0.9460	Brown	Brown (0.6169)	0.7410	F-III	F-III (0.9996)	0.7833
82	Brown	Brown (0.7758)	0.9460	Blond	Brown (0.6294)	0.7410	F-I	F-III (0.5864)	0.7833
83	Brown	Brown (0.9978)	0.9460	Black	Black (0.6120)	0.6120	F-IV	F-V (0.8312)	0.9934
88	Brown	Brown (0.9945)	0.9460	Black	Black (0.8595)	0.8592	F-III	F-III (0.9216)	0.7833
89	Intermediate	Brown (0.5474)	0.9460	Brown	Brown (0.6221)	0.7410	F-II	F-III (0.9999)	0.7833
91	Brown	Brown (0.9972)	0.9460	Black	Black (0.7683)	0.8592	F-III	F-III (0.9981)	0.7833
96	Brown	NA	0.9460	Black	Black (0.8780)	0.8592	F-II	F-III (0.9965)	0.7833
98	Brown	Brown (0.7667)	0.9460	Brown	Brown (0.5531)	0.7410	F-II	F-III (0.6326)	0.7833
103	Brown	Brown (0.9972)	0.9460	Black	Black (0.7915)	0.8592	F-IV	F-III (0.9981)	0.7833
104	Brown	Brown (0.9857)	0.9460	Black	Black (0.5666)	0.8592	F-III	F-III (0.9998)	0.7833
106	Brown	Brown (0.9930)	0.9460	Brown	Brown (0.5052)	0.7410	F-III	F-III (0.9952)	0.7833
107	Brown	Brown (0.9377)	0.9460	Red	Red (0.8363)	0.9289	F-II	F-III (0.5412)	0.7833
109	Brown	Brown (0.9822)	0.9460	Black	Brown (0.5415)	0.7410	F-III	F-III (0.9989)	0.7833
122	Brown	NA	0.9460	Black	Brown (0.4889)	0.7410	F-III	F-III (0.89117)	0.7833
123	Brown	Brown (0.9930)	0.9460	Brown	Brown (0.7204)	0.7410	F-II	F-III (0.8211)	0.7833
124	Brown	NA	0.9460	Black	Black (0.4559)	0.8592	F-III	F-III (0.9968)	0.7833
134	Brown	Brown (0.9426)	0.9460	Blond	Brown (0.4957)	0.7410	F-I	F-III (0.9999)	0.7833
144	Brown	Brown (0.9978)	0.9460	Black	Black (0.8555)	0.8592	F-IV	F-III (0.9377)	0.7833
145	Brown	Blue (0.8478)	0.9460	Black	Blond (0.7053)	0.8132	F-III	F-II (0.6178)	0.7627
147	Brown	Brown (0.9565)	0.9460	Black	NA	0.9289	F-III	F-III (0.9506)	0.7833
151	Brown	Brown (0.9946)	0.9460	Black	Brown (0.5491)	0.7410	F-II	F-III (0.8482)	0.7833
152	Brown	Brown (0.9815)	0.9460	Brown	Brown (0.6806)	0.7410	F-II	F-III (0.8618)	0.7833
153	Intermediate	NA	0.9460	Brown	Black (0.7481)	0.8592	F-III	F-III (0.8618)	0.7833
154	Brown	NA	0.9460	Black	Black (0.5126)	0.8592	F-III	F-III (0.9969)	0.7833
155	Brown	Blue (0.9150)	0.9460	Black	Blond (0.6323)	0.8132	F-II	F-III (0.9991)	0.7833
156	Brown	NA	0.9460	Brown	Brown (0.3984)	0.7410	F-II	F-III (0.9780)	0.7833
159	Blue	Brown (0.9767)	0.9460	Blond	Black (0.5478)	0.8592	F-I	F-III (0.9980)	0.7833
160	Brown	Brown (0.7667)	0.9460	Black	Brown (0.5418)	0.7410	F-III	F-III (0.9389)	0.7833
158	Brown	Brown (0.9780)	0.9460	Brown	Brown (0.6362)	0.7410	F-III	F-III (0.9559)	0.7833
254	Intermediate	Blue (0.6498)	0.9460	Brown	Brown (0.5865)	0.7410	F-III	F-III (0.7663)	0.7833
303	Brown	NA	0.9460	Brown	Brown (0.5604)	0.7410	F-III	F-V (0.6829)	0.9934

## Data Availability

The genotype dataset reported in this paper is reported as Supplementary Materials.

## Supplementary Materials

The following supporting information can be downloaded at: https://www.mdpi.com/article/10.3390/genes17030354/s1, Table S1: Genotype and sequencing quality data for the 107 SNPs included in the ForenSeq IMAGEN Kit (Illumina), used for the prediction of eye, hair, and skin color, as well as biogeographic ancestry, were analyzed in 41 samples from Mexican individuals.
